# Expression of Concern: Observations of linear aggregation behavior in rotifers (*Brachionus calyciflorus*)

**DOI:** 10.1371/journal.pone.0325957

**Published:** 2025-06-10

**Authors:** The PLOS One Editors

The ***PLOS One*** Editors issue this Expression of Concern to inform readers that issues came to light which call into question the reliability and/or provenance of the research reported in this article [1]. Specifically,

- This article [1] was identified as one of a series of submissions for which we have concerns about similarities across articles from different author groups.
- The Editors are concerned about aspects of this article’s peer review process.
