# Supplementary material for: Observations of linear aggregation behavior in rotifers (Brachionus calyciflorus)
Source: PLoS One. 2021 Aug 19;16(8):e0256387. doi: 10.1371/journal.pone.0256387 (PMC8376037; doi:10.1371/journal.pone.0256387)
Supplement: Attachment — Submitted filename: Response to Reviewers.docx [file pone.0256387.s002.docx]

Dear Editor,

Thank you for carefully reviewing our manuscript previously titled “**Observations of linear aggregation Behavior in Rotifers (*Brachionus calyciflorus*)**” for possible publication in the ***PLOS ONE***. We are grateful to you and your reviewers for their constructive critique. We have revised the manuscript, highlighting our revisions in red and have attached point-by-point responses detailing how we have revised the manuscript in response to the reviewers' comments below.

We added reviewe of rotifer behaviors, and cited some latest references, and corrected various formatting issues, spelling and grammatical mistakes e.g. and arranged the references correctly.

Thank you for your consideration and further review of our manuscript. Please do not hesitate to contact us with any further questions or recommendations.

Yours Sincerely,

shuanghuaicheng2018@hainmc.edu.cn (SC); yanshijiao@hainmc.edu.cn (SY); yunxiazhang@hainmc.edu.cn (YZ); lvchuanzhu677@126.com (CL).

Reviewer Comments:

Reviewer #1: The manuscript entitled "Observations of linear aggregation Behavior in Rotifers (*Brachionus calyciflorus*)" discusses linear aggregation in *Brachionus calyciflorus* upon increase of density and such behavioral pattern among asocial animal like rotifers is an interesting finding. The manuscript may be accepted with minor corrections. The authors need to correct various formatting issues, spelling and grammatical mistakes e.g. " mL-1" etc. Similarly, references also need formatting and more latest references should be included such as Nature, 2017, 551,313 on bacterial quorum sensing etc.

**Response:**

Thank you for your reviewing of our article and giving suggestions. We have modified it according to your suggestions and submitted the revised manuscript. Including the spelling of words, grammar mistakes, the format of the references and so on. And add some latest references including such as Nature, 2017, 551,313 on bacterial quorum sensing etc.

Reviewer #2: The manuscript entitled "Observations of linear aggregation Behavior in Rotifers (*Brachionus calyciflorus*)" discusses previously undocumented, linear aggregation in rotifer *Brachionus calyciflorus* in response to increased culture density. The novel findings are well documented and presented. The manuscript may be accepted with few minor corrections. The author shall review and correct various formatting, grammatical and spelling errors and arrange the references correctly.

**Response:**

Thank you for your reviewing of our article and giving suggestions. We have modified it according to your suggestions and submitted the revised manuscript. Including the spelling of words, grammar mistakes, the format of the references and so on.
